# Chronic disease risk and protective behaviors in Brazilian state capitals and the Federal District, according to the National Health Survey and the Chronic Disease Risk and Protective Factors Telephone Survey Surveillance System, 2019

**DOI:** 10.1590/SS2237-9622202200009.especial

**Published:** 2022-08-08

**Authors:** Thaís Cristina Marquezine Caldeira, Marcela Mello Soares, Luiza Eunice Sá da Silva, Izabella Paula Araújo Veiga, Rafael Moreira Claro

**Affiliations:** 1 Universidade Federal de Minas Gerais, Programa de Pós-graduação em Saúde Pública, Belo Horizonte, MG, Brazil; 2 Universidade Federal de Minas Gerais, Programa de Pós-graduação em Nutrição e Saúde, Belo Horizonte, MG, Brazil; 3 Universidade Federal de Minas Gerais, Departamento de Nutrição, Belo Horizonte, MG, Brazil

**Keywords:** Health Surveys, Chronic Disease, Lifestyle, Risk Factors, Epidemiological Monitoring, Cross-sectional Studies

## Abstract

**Objective:**

To describe and compare the results of the main risk and protective factors for chronic non-communicable diseases, in the 26 Brazilian capitals and the Federal District, obtained through the National Health Survey (PNS) and the Surveillance System for Risk and Protective Factors for Chronic Diseases by Telephone Survey (VIGITEL) in 2019.

**Methods:**

Cross-sectional study, in which the difference in prevalence between health behavior indicators investigated by PNS and VIGITEL was calculated.

**Results:**

The largest discrepancy between the surveys, PNS (n = 32,111) and VIGITEL (n = 52,443), were observed in relation to leisure-time physical activity (6.8 in percentage points - p.p.), recommended physical activity in the transport domain (7.4 p.p.), and high screen time (21.8 p.p.). Both surveys presented similar prevalence regarding nutritional status, food consumption, smoking, alcohol abuse and negative self-rated health.

**Conclusion:**

Prevalence in both surveys presented small differences, but point to results in the same direction.

**Study contributions**

**Main results**

Both surveys show similar prevalence for most of the indicators. Significant differences were observed for the indicators of physical activity and sedentary behavior.

**Implications for services**

The comparison between surveys carried out through different methodologies enables the identification of the limits of their application in public policies proposals and monitoring.

**Perspectives**

The results contribute to the improvement of surveys and indicators used to monitor risk and protective factors for chronic diseases in the country.

## Introduction

Chronic non-communicable diseases (NCDs) are the major public health problem at present because they result in loss of quality of life and a high number of deaths.^1^ ^,^ ^2^ The risk factors involved in the etiology of these diseases stand out for being behavioral and modifiable, such as: inadequate diet, physical inactivity, abusive consumption of alcoholic beverages and smoking.^1^

In this scenario, it is worth highlighting the importance of surveillance and monitoring of these diseases and their risk factors for the planning and promotion of health in the population.^3^ The information obtained through population health surveys is essential to understand the health profile of the population and the distribution of risk factors.^4^ Such researches have been carried out in Brazil since the 1970s, mainly through household surveys.^5^ ^,^ ^6^ Given the high cost and logistics involved in household researches, the use of telephone interviews (faster and at a lower cost) made it possible to carry out health surveys capable of continuously detecting changes in determining and conditioning factors of the population’s health.^7^

Since 2006, an annual health survey has been carried out in Brazil, using landline telephones, aiming at the continuous monitoring of the prevalence and distribution of the most relevant determinants associated with NCDs. The Surveillance System for Risk and Protective Factors for Chronic Diseases by Telephone Survey (VIGITEL), implemented by the Ministry of Health, together with other surveys carried out in the country, enable the expansion of knowledge on the Brazilian population's health status.^7^ ^,^ ^8^

Even so, household interviews are a widely used method in the investigation of health outcomes in Brazil. Carried out for the first time in 2013, by means of face-to-face interviews and with a broad scope, the National Health Survey (PNS) took place again in 2019, with the objective of collecting and updating information regarding the living conditions and health status of the population.^5^

Given the advantages of using telephone interviews for the continuous provision of health data concerning the population and the robustness of a household survey, the comparison between data obtained from two health surveys carried out in the same year enables the ensures the reliability the information collected in the country. Thus, the objective of this study was to describe and compare the results of the main risk and protective factors for NCDs, in the 26 Brazilian capitals and the Federal District, obtained through the PNS and VIGITEL, in 2019. 

## Methods

This was a cross-sectional study, carried out using data from two large Brazilian population surveys, the PNS and VIGITEL, in 2019. 

The PNS is a population-based household survey, with national representation, carried out by the Ministry of Health and the Brazilian Institute of Geography and Statistics (IBGE), with the objective of producing data on the population's health and living conditions. The PNS 2019 sampling process was based on the master sample for the IBGE's integrated household survey system.^9^ From that sample, a cluster sampling process was established, starting with the census sectors (called primary sampling units), followed by a simple random sample of permanent households (secondary units) and, finally, a resident aged ≥ 15 years was randomly selected for the interview (tertiary unit).^9^ The interviews were collected between August 2019 and March 2020.^9^ For the present study, a subsample referring to individuals aged ≥ 18 years residing in the 26 state capitals and the Federal District was used, in order to enable comparison with data from VIGITEL.

VIGITEL is a telephone survey carried out annually by the Ministry of Health, starting in 2006, with the objective of monitoring the main risk and protective factors for NCDs. In each edition of VIGITEL, a simple random sample of adults ≥ 18 years of age residing in households that have at least one landline, in the 26 Brazilian capitals and the Federal District, is investigated. The sampling used establishes around 2,000 interviews per year in each city, allowing for the estimation of all the factors surveyed with a maximum error of 2 percentage points (p.p) and a 95% confidence interval (95%CI). Smaller samples, of about 1,500 interviews, are accepted in cities where the landline telephone service covers less than 40% of the households, in which case maximum errors of 3 p.p are accepted.^8^ For the present study, data collected from January to December 2019 were used. 

Weighting factors are assigned to data from both surveys in order to adjust for “non-response” and to ensure that the data represent the universe of the target population [equating their sex and age distribution to that of the total population, in the case of the PNS; and sex, six age groups in years (18-24, 25-4, 35-44, 45-54, 55-64 and ≥ 65)and three levels of schooling in years of study (0-8, 9-11 and ≥ 12), in the case of VIGITEL]. More information on the PNS and VIGITEL methodology can be found in specific publications.^8^ ^,^ ^9^

Initially, the survey questionnaires were collated so that comparable indicators could be identified. To this end, the module on lifestyles (module P) of the PNS was compared to a full version of the 2019 VIGITEL questionnaire, since both questionnaires were developed to enable the creation of indicators involving the same theme. As a result, the comparison between the instruments turned to the analysis of the statements and response options, in order to promote the comparison only for indicators whose expected comparability were, at least, satisfactory. At the end of this process, indicators were selected referring to nutritional status (risk factors: self-reported obesity and overweight), dietary intake (protective factor: consumption of unprocessed or minimally processed foods; and risk factor: consumption of ultra-processed foods), physical activity and sedentary behavior (protective factors: recommended physical activity during leisure time and transport; and risk factor: high screen time), smoking (risk factor: current smoker), alcohol consumption (risk factor: alcohol abuse) and perceived health status (risk factor: negative self-assessment of health status). A detailed description of the questions involved in each indicator, in each of the surveys, is presented in Box 1.

**Box 1:** Questions and indicators of the National Health Survey and the Surveillance System for Risk and Protective Factors for Non-Communicable Diseases (NCDs) by Telephone Survey (NCDs), 2019

Risk or protective factor for NCDs	Questions		Indicator
	PNS^a^ 2019	VIGITEL^b^ 2019	
**Nutricional status**			
Percentage of adults with overweight^c^	*Do you know how much you weigh?* [answer in kilograms]; *Do you know how tall you are?* [answer in centimeters].	*Do you know how much you weigh (even if it is an approximate value)?* [answer in kilograms]; *Do you know how tall you are?* [answer in meters].	Number of overweight individuals/number of individuals interviewed. Individuals with a body mass index (BMI) ≥ 25 kg/m^2^, calculated based on the person’s weight in kilograms divided by the square of their height in meters, both self-reported, were considered overweight.
Percentage of adults with obesity^c^	*Do you know how much you weigh?* [answer in kilograms] and *Do you know how tall you are?* [answer in centimeters].	*Do you know how much you weigh (even if it is an approximate value)?* [answer in kilograms]. *Do you know how tall you are?* [answer in meters].	Number of obese individuals/number of individuals interviewed. Individuals with a body mass index (BMI) ≥ 30 kg/m^2^, calculated based on the person’s weight in kilograms divided by the square of their height in meters, both self-reported, were considered obese.
**Dietary intake**			
Percentage of adults who consumed five or more non- or minimally processed food groups that are protective for chronic diseases on the day before the interview^d^	Now let's talk about your diet. I'm going to ask you some questions about foods you ate yesterday. Let's start with natural or basic foods. *Yesterday, did you eat: a. Rice, pasta, polenta, couscous or corn?; b. Common potato, manioc/cassava or yam?; c. Beans, peas, lentils or chickpeas?; d. Beef, pork, chicken or fish?; and. Egg (fried, boiled or scrambled)?; f. Lettuce, kale, broccoli, watercress or spinach?; h. Pumpkin, carrot, sweet potato or okra?; i. Tomato, cucumber, zucchini, eggplant, chayote or beetroot?; j. Papaya, mango, yellow melon or pequi?; k. Orange, banana, apple or pineapple?; l. Milk?; m. Peanuts, cashews or brazil nuts/pará nuts?* (yes; no).	Now I'm going to list some foods and I would like you to tell me if you ate any of them yesterday (from the moment you woke up until you went to sleep). I'll start with natural or basic foods: lettuce, kale, broccoli, watercress or spinach; pumpkin, carrot, sweet potato or okra; papaya, mango, yellow melon or pequi; tomato, cucumber, zucchini, eggplant, chayote or beetroot; orange, banana, apple or pineapple; beans, peas, lentils or chickpeas; peanuts, cashews or brazil nuts/pará nuts (yes; no).	Number of individuals who consumed five or more groups of non- or minimally processed protective foods for chronic diseases on the list, on the day before the interview/number of individuals interviewed.
**Dietary intake**			
Percentage of adults who consumed five or more ultra-processed food groups the day before the interview^c^	*Yesterday, did you consumed: a. soft drink?; b. canned or carton fruit juice or powdered juice?; c. chocolate drink or flavored yogurt?; d. packaged snacks or crackers/saltines?; and biscuits/cookies, or sandwich cookies or packet cake?; f. ice cream, chocolate, flan or other industrialized desserts?; g. sausage, bologna or ham?; h. sliced bread, hot-dog bun or hamburger bun?; i. margarine, mayonnaise, ketchup or other industrialized sauces?; j. instant noodles, packet soup, frozen lasagna or any other premade, processed frozen meal?* (yes; no).	Now I'm going to list some foods and I would like you to tell me if you ate any of them yesterday (from the moment you woke up until you went to sleep). Now I will list industrialized foods or products: soft drinks; canned or carton fruit juice; powdered juice; chocolate drink; flavored yogurt; packaged snacks (or chips) or crackers/saltines; biscuits/cookies, sandwich cookies or packet cake; chocolate, ice cream, gelatin, flan or any other industrialized dessert; sausage, bologna or ham; sliced bread, hot-dog bun or hamburger bun; mayonnaise, ketchup or mustard; margarine; instant noodles, packet soup, frozen lasagna or any other premade, processed frozen meal (yes; no).	Number of individuals who consumed five or more groups of ultra-processed foods on the day before the interview/number of individuals interviewed.
**Physical activity and sedentary behavior**			
Percentage of adults who engage in free-time physical activity equivalent to at least 150 minutes of moderate intensity activity per week^d^	I*n the past twelve months, have you done any type of physical activity or practiced any sports?* (do not consider physical therapy) (yes; no). *How many days a week do you usually do (or used to do) a physical activity or practice a sport?* [answer in number of days]. *In general, on the day that you do (did) a physical activity or practiced a sport, how long did that activity last?* [answer in hours/minutes]. *Which physical activity do you do (or did) or which sport do you practice (or practiced) most often?* [physical activity or sport option].	*In the past three months, have you done any type of physical activity or practiced any sport?* (yes; no). *What is the main type of physical activity you did or sport that you practiced?* [physical activity or sports practice option]. *Do you exercise at least once a week?* (yes; no). *How many days a week do you usually do physical activities or practice a sport?* (1 to 2 days a week; 3 to 4 days a week; 5 to 6 days a week; every day (including Saturday and Sunday). *On the day you exercise or practice a sport, how long does this activity last?* (Less than 10 minutes; from 10 to 19 minutes; from 20 to 29 minutes; from 30 to 39 minutes; from 40 to 49 minutes; from 50 to 59 minutes; 60 minutes or more).	Number of individuals who engage in moderate intensity physical activity for at least 150 minutes a week or in vigorous intensity physical activity for at least 75 minutes a week/number of individuals interviewed. Activities that last less than 10 minutes are not considered for the purpose of calculating the daily sum of minutes spent by the individual with physical activities. Walking, treadmill walking, bodybuilding, water aerobics, gymnastics in general, swimming, martial arts and fighting, cycling, volleyball/foot volley and dance were classified as moderate intensity practices; running, treadmill running, aerobics, soccer/futsal, basketball and tennis were classified as vigorous intensity practices.
**Physical activity and sedentary behavior**			
Percentage of adults who engage in physical activity during transport equivalent to at least 150 minutes of moderate intensity activity per week^d^	*To travel to or from work, do you walk or cycle?* (yes; no); *How many days a week do you walk or cycle?* [answer in number of days]. *How much time do you spend, per day, to travel this route on foot or by bicycle, considering going to and from work?* [answer in hours/minutes]. *In your usual activities (such as going to a course, school or club or taking someone to a course, school or club), how many days a week do you do any activity that involves walking or cycling?* (Except work) [answer in number of days]. *On the day you do those activities, how much time do you spend on foot or by bicycle, considering the round trip?* [answer in hours/minutes].	*Do you walk or cycle to or from your work?* (yes; yes, part of the route; no). *How much time do you spend to go to and from this place (on foot or by bicycle)?* (less than 10 minutes; from 10 to 19 minutes; from 20 to 29 minutes; from 30 to 39 minutes; from 40 to 49 minutes; from 50 to 59 minutes; 60 minutes or more). *Are you currently attending a course/school or taking someone to a course/school?* (yes; no). *To go to or to return from this course/school, do you do any walking or cycling?* (yes; yes, part of the route; no). *How much time do you spend to go to and from this place (on foot or by bicycle)?* (Less than 10 minutes; from 10 to 19 minutes; from 20 to 29 minutes; from 30 to 39 minutes; from 40 to 49 minutes; from 50 to 59 minutes; 60 minutes or more).	Number of individuals who walk or bike to work or school and who spend at least 30 minutes per day travelling back and forth/number of individuals interviewed. Questions related to transport to work and/or school and/or a course are considered.
**Physical activity and sedentary behavior**			
Percentage of adults who spend 3 or more hours of free time watching television or using a computer, tablet or cell phone^c^	*On average, how many hours a day do you spend watching television?* (less than 1 hour; from 1 hour to less than 2 hours; from 2 hours to less than 3 hours; from 3 hours to less than 6 hours; 6 hours or more; I do not watch television); *On a single day, how many hours of your free time (excluding work) do you usually spend using a computer, tablet or cell phone for leisure activities such as: using social networks, watching the news, watching videos, playing games, etc.?* (Less than 1 hour; from 1 hour to less than 2 hours; from 2 hours to less than 3 hours; from 3 hours to less than 6 hours; 6 hours or more; I do not usually use a computer, tablet or cell phone in my free time).	*On average, how many hours a day do you usually spend watching television?* (Less than 1 hour; from 2 to 3 hours; from 3 to 4 hours; from 4 to 5 hours; from 5 to 6 hours; more than 6 hours; I do not watch television); *On average, how many hours of your free time (excluding work) do you spend using a computer, tablet or cell phone per day?* (Less than 1 hour; from 2 to 3 hours; from 3 to 4 hours; from 4 to 5 hours; from 5 to 6 hours; more than 6 hours).	Number of individuals who report the habit of watching television or using the computer, tablet or cell phone for three or more hours a day/number of individuals interviewed.
**Smoking and heavy episodic drinking**			
Percentage of smokers^c^	*Do you currently smoke any tobacco product?* (yes, daily; yes, less than daily; I don’t currently smoke).	*Do you currently smoke?* (yes, daily; yes, but not daily; no).	Number of smoking individuals/number of individuals interviewed. The individual who answered positively to the question ‘*Do you currently smoke?*’ was considered a smoker, regardless of the number of cigarettes, frequency and duration of the smoking habit.
**Smoking and heavy episodic drinking**			
Percentage of adults who engaged in heavy episodic drinking^c^	*In the past thirty days, have you consumed five or more drinks containing alcohol on one occasion?* (One alcoholic drink is equivalent to one can of beer, one glass of wine, one dose of liquor, whisky or any other distilled alcoholic beverage). (yes; no).	*In the past thirty days, have you consumed five/four (for men/women) or more drinks of alcohol on one occasion?* (yes; no).	Number of adults who consumed alcohol abusively/number of individuals interviewed. Abusive consumption of alcoholic beverages was considered to be five or more drinks (men) or four or more drinks (women) on a single occasion, at least once in the last 30 days.
**Self-rated health status**			
Percentage of adults who negatively rated their health status^c^	*In general, how do you rate your health?* (very good; good; fair; bad; very bad).	*Would you classify your health status as: very good, good, fair, bad or very bad?* (very good; good; fair; bad; very bad).	Number of adults who rated their health status as bad or very bad/number of individuals interviewed.

a) PNS: National Health Survey; b) VIGITEL: Surveillance System for Risk and Protective Factors for Chronic Diseases by Telephone Survey; c) Risk Factor; d) Protective factor.

To enable comparison between surveys, sociodemographic data were also analyzed, such as sex (male and female) and age (distributed into ranges: 18-34 years, 35-54 years and ≥ 55 years) of the individuals interviewed in each survey. 

The prevalence of each of the indicators (and their 95%CI) was then independently estimated for each of the surveys. This procedure was carried out for the entire population, by sex and age group. Differences in estimated prevalence were identified through absolute difference (in p.p.) and relative difference (in percentage) between the indicators of both surveys. Stata software, version 14.2, was used to organize, process and analyze the data. All analyses were performed using the survey module, taking the sample design of each of the surveys into consideration.

VIGITEL databases are available on the official website of the Ministry of Health (http://svs.aids.gov.br/download/VIGITEL/; accessed in: December 2020). The conduction of VIGITEL was approved by the National Committee of Ethics in Research on Human Beings (Conep), under opinion No. 65610017.1.0000.0008. PNS data are available on IBGE’s official website (https://www.ibge.gov.br/estatisticas/sociais/saude.html; accessed in: December 2020). The conduction of the PNS was approved by Conep under opinion No. 3.529.376. For both surveys, the Free and Informed Consent Term was obtained at the time of the interview.

## Results

Data from 32,111 adults living in the capitals and the Federal District who were interviewed by the PNS and 52,443 adults interviewed by VIGITEL, both in 2019, were included in the study. The largest part of the population living in the capitals and the Federal District interviewed by the PNS was female (54.9%), with the highest proportion in the total of adults between 35 and 54 years of age (37.2%). Among the adults interviewed by VIGITEL, the female population was also the majority (54.0%), and the highest proportion in the total of adults aged 18 to 34 years (38.8%) (Table 1).

**Table 1:** Prevalence and 95% confidence interval of the adult population in the capitals of the 26 states and the Federal District, by sex and age, according to the National Health Survey and the Surveillance System for Risk and Protective Factors for Chronic Diseases by Telephone Survey, Brazil, 2019

Variables	PNS^a^ 2019		VIGITEL^b^ 2019	
	n = 32,111		n = 52,443	
	%	95%CI^c^	%	95%CI^c^
**Male**				
18 to 34 years	35.2	33.7;36.7	43.4	41.8;45.0
35 to 54 years	37.5	36.2;38.8	35.4	33.9;36.8
≥ 55 years	27.4	26.1;28.6	21.3	20.3;22.3
Total	45.1	44.2;46.0	46.0	45.0;46.9
**Female**				
18 to 34 years	30.1	28.8;31.4	34.9	33.7;36.2
35 to 54 years	37.1	35.9;38.2	37.9	36.8;39.0
≥ 55 years	32.8	31.7;34.0	27.2	26.3;28.0
Total	54.9	54.0;55.8	54.0	53.1;55.0
**Total**				
18 to 34 years	32.4	31.3;33.4	38.8	37.8;39.8
35 to 54 years	37.2	36.4;38.1	36.7	35.8;37.6
≥ 55 years	30.4	29.5;31.3	24.5	23.8;25.1

a) PNS: National Health Survey; b) VIGITEL: Surveillance System for Risk and Protective Factors for Chronic Diseases by Telephone Survey; c) 95%CI: 95% confidence interval.

Regarding the indicators studied, the greatest percentage differences were observed in the prevalence of recommended physical activity during leisure time, with the prevalence estimated from VIGITEL exceeding 6.8 p.p. to that of the PNS (PNS = 32.2% vs. VIGITEL = 39.0%). Values higher than those identified in VIGITEL were observed in the PNS for recommended transport physical activity, a difference of 7.4 p.p. (PNS = 21.5% vs. VIGITEL = 14.1%), and for high screen time, a difference of 21.8 p.p. (PNS = 84.5% vs. VIGITEL = 62.7%). In addition, the prevalence values of overweight, obesity, consumption of unprocessed or minimally processed foods, consumption of ultra-processed foods, smokers, abusive alcohol consumption and negative self-assessment of health status were similar in both surveys (Table 2).

**Table 2:** Prevalence, 95% confidence interval and differences between the selected risk and protective factors for chronic non communicable diseases (NCDs), in the adult population of the capitals of the 26 states and the Federal District according to the National Health Survey and the Surveillance System for Risk and Protective Factors for Chronic Diseases by Telephone Survey, Brazil, 2019

Risk and protective factors for NCDs	PNS^a^ 2019		VIGITEL^b^ 2019		Diff.^h^
	n = 32,111		n = 52,443		
	%	95%CI^c^	%	95%CI^c^	p.p. (%)^i^
**Nutritional status**					
Overweight	58.5	57.6;59.4	55.3	54.4;56.3	3.2 (5.5)
Obesity	20.5	19.8;21.2	20.3	19.5;21.0	0.2 (0.8)
**Dietary intake**					
Consumption of unprocessed or minimally processed foods^d^	29.7	28.7;30.6	29.8	28.9;30.6	-0.1 (-0.2)
Consumption of ultra-processed foods^e^	16.7	15.9;17.5	18.2	17.4;19.0	-1.5 (-9.1)
**Physical activity and sedentarism**					
Physical activity during leisure time^f^	32.2	34.3;36.2	39.0	38.0;39.9	-6.8 (-21.1)
Physical activity during transport^f^	21.5	20.6;22.4	14.1	13.4;14.9	7.4 (34.2)
High screen time^g^	84.5	83.8;85.2	62.7	61.8;63.6	21.8 (25.8)
**Smoking and harmful alcohol consumption**					
Smokers	11.4	10.8;12.0	9.8	9.2;10.5	1.6 (13.7)
Harmful alcohol consumption	19.2	18.4;20.0	18.8	18.0;19.6	0.4 (2.0)
**Perceived health status**					
Negative self-assessment of health	4.4	4.1;4.8	4.8	4.4;5.2	-0.4 (-9.5)

a) PNS: National Health Survey; b) VIGITEL: Surveillance System for Risk and Protective Factors for Chronic Diseases by Telephone Survey; c) 95%CI: 95% confidence interval; d) ≥ 5 unprocessed or minimally processed foods in the 24 hours prior to the interview; e) ≥ 5 ultra-processed foods in the 24 hours prior to the interview; f) ≥ 150 minutes per week; g) ≥ 3 hours per day; h) Difference between the PNS and VIGITEL data; i) Absolute difference (in percentage points [p.p.]) and relative difference (in percentage) using the PNS data as the baseline.

In the analysis stratified by sex, there was a difference of 2.4 p.p. (PNS = 29.9% vs. VIGITEL = 32.3%) for females, between the prevalence of consumption of unprocessed or minimally processed foods. All differences referring to physical activity and physical inactivity were similar to the results of the total population for males, while only results referring to transport and high screen time remained similar for females (Table 3).

**Table 3:** Prevalence, 95% confidence interval and differences between the selected risk and protective factors for chronic non communicable diseases (NCDs), in the adult population of the capitals of the 26 states and the Federal District according to sex, for the National Health Survey and the Surveillance System for Risk and Protective Factors for Chronic Diseases by Telephone Survey, Brazil, 2019

Risk and protective factors for NCDs	PNS^a^ 2019		VIGITEL^b^ 2019		Diff.^h^
	n = 32,111		n = 52,443		
	%	95%CI^c^	%	95%CI^c^	p.p. (%)^i^
**Male**					
**Nutritional status**					
Overweight	61.8	60.5;63.7	57.1	55.6;59.0	4.7 (7.6)
Obesity	19.2	18.3;20.3	19.5	18.3;20.6	-0.3 (-1.6)
**Dietary intake**					
Consumption of unprocessed or minimally processed foods^d^	29.5	28.2;30.7	26.9	25.6;28.2	2.6 (8.9)
Consumption of ultra-processed foods^e^	19.3	18.1;20.5	21.8	20.5;23.2	-2.5 (-13.2)
**Physical activity and sedentarism**					
Physical activity during leisure time^f^	41.2	39.8;42.6	46.7	45.2;48.3	-5.5 (-13.4)
Physical activity during transport^f^	22.5	21.3;23.7	14.5	13.4;15.7	8.0 (35.4)
High screen time^g^	83.7	82.5;84.7	63.9	62.4;65.3	19.8 (23.7)
**Smoking and harmful alcohol consumption**					
Smokers	14.4	13.4;15.4	12.3	11.2;13.5	2.1 (14.4)
Harmful alcohol consumption	28.3	26.9;29.6	25.3	24.0;26.7	3.0 (10.4)
**Perceived health status**					
Negative self-assessment of health	3.4	3.0;3.9	3.4	2.9;3.9	0.0 (0.7)
**Female**					
**Nutritional status**					
Overweight	55.7	54.5;56.9	53.9	52.7;55.2	1.8 (3.2)
Obesity	21.5	20.6;22.5	21.0	20.0;21.9	0.5 (2.3)
**Dietary intake**					
Consumption of unprocessed or minimally processed foods^d^	29.9	28.7;31.1	32.3	31.2;33.3	-2.4 (-7.9)
Consumption of ultra-processed foods^e^	14.5	13.6;15.4	15.1	14.2;16.1	-0.6 (-4.4)
**Physical activity and sedentarism**					
Physical activity during leisure time^f^	30.4	29.1;31.6	32.4	31.3;33.5	-2.0 (-6.5)
Physical activity during transport^f^	20.7	19.6;21.8	13.8	12.9;14.7	6.9 (33.2)
High screen time^g^	85.3	84.4;86.1	61.7	60.5;62.8	23.6 (27.7)
**Smoking and harmful alcohol consumption**					
Smokers	8.9	8.3;9.6	7.7	7.1;8.4	1.2 (13.2)
Harmful alcohol consumption	11.8	11.0;12.5	13.3	12.4;14.2	-1.5 (-12.3)
**Perceived health status**					
Negative self-assessment of health	5.3	4.8;5.8	6.0	5.4;6.6	-0.7 (-13.3)

a) PNS: National Health Survey; b) VIGITEL: Surveillance System for Risk and Protective Factors for Chronic Diseases by Telephone Survey; c) 95%CI: 95% confidence interval; d) ≥ 5 unprocessed or minimally processed foods in the 24 hours prior to the interview; e) ≥ 5 ultra-processed foods in the 24 hours prior to the interview; f) ≥ 150 minutes per week; g) ≥ 3 hours per day; h) Difference between the PNS and VIGITEL data; i) Absolute difference (in percentage points [p.p.]) and relative difference (in percentage) using the PNS data as the baseline.

In the age group from 18 to 34 years old, the greatest differences were observed in relation to the prevalence of consumption of non- or minimally processed foods, at 3.5 p.p. (PNS = 21.9% vs. VIGITEL = 25.4%), leisure time physical activity, at 4.9 p.p. (PNS = 43.9% vs. VIGITEL = 48.8%), of physical activity during transport, at 8.0 p.p. (PNS = 23.2% vs. VIGITEL = 15.2%), high screen time, with a difference of 13.2 p.p. (PNS = 88.6% vs. VIGITEL = 75.4%), of smokers, at 3.2 p.p. (PNS = 12.0% vs. VIGITEL = 8.8%) and negative self-assessment of health status, at 2.2 p.p. (PNS = 1.9% vs. VIGITEL = 4.1%) (Table 4).

**Table 4:** Percentage, 95% confidence interval and differences between the selected risk and protective factors for chronic non communicable diseases (NCDs), in the adult population of the capitals of the 26 states and the Federal District according to age, for the National Health Survey and the Surveillance System for Risk and Protective Factors for Chronic Diseases by Telephone Survey, Brazil, 2019

Risk and protective factors for NCDs	PNS^a^ 2019		VIGITEL^b^ 2019		Diff.^h^
	n = 32,111		n = 52,443		
	%	95%CI^c^	%	95%CI^c^	p.p. (%)^i^
**18 to 34 years**					
**Nutritional status**					
Overweight	46.5	44.7;48.3	44.9	43.1;46.8	1.6 (3.4)
Obesity	15.6	14.4;16.8	15.5	14.2;16.9	0.1 (0.5)
**Dietary intake**					
Consumption of unprocessed or minimally processed foods^d^	21.9	20.5;23.3	25.4	23.9;27.0	-3.5 (-16.2)
Consumption of ultra-processed foods^e^	25.1	23.6;26.6	25.6	24.0;27.2	-0.5 (-2.0)
**Physical activity and sedentarism**					
Physical activity during leisure time^f^	43.9	42.2;45.6	48.8	47.0;50.7	-4.9 (-11.2)
Physical activity during transport^f^	23.2	21.6;24.9	15.2	13.8;16.6	8.0 (34.6)
High screen time^g^	88.6	87.6;89.7	75.4	73.8;77.0	13.2 (14.9)
**Smoking and harmful alcohol consumption**					
Smokers	12.0	10.8;13.3	8.8	7.6;10.0	3.2 (26.7)
Harmful alcohol consumption	25.7	24.0;27.3	26.1	24.4;27.8	-0.4 (-1.7)
**Perceived health status**					
Negative self-assessment of health	1.9	1.4;2.4	4.1	3.4;4.9	-2.2 (-117.0)
**5 to 54 years**					
**Nutritional status**					
Overweight	65.1	63.8;66.4	62.3	60.8;63.7	2.8 (4.2)
Obesity	23.6	22.5;24.7	23.6	22.3;24.9	0.0 (0.0)
**Dietary intake**					
Consumption of unprocessed or minimally processed foods^d^	30.7	29.3;32.1	31.6	30.3;33.0	-0.9 (-3.1)
Consumption of ultra-processed foods^e^	15.4	14.3;16.4	16.6	15.4;17.8	-1.2 (-8.0)
**Physical activity and sedentarism**					
Physical activity during leisure time^f^	34.5	33.1;36.0	35.8	34.4;37.2	-1.3 (-3.8)
Physical activity during transport^f^	21.7	20.4;23.0	16.9	15.7;18.0	4.8 (22.1)
High screen time^g^	79.9	78.7;81.1	58.3	56.8;59.7	21.6 (27.1)
**Smoking and harmful alcohol consumption**					
Smokers	11.4	10.5;12.3	10.3	9.2;11.3	1.1 (10.0)
Harmful alcohol consumption	20.8	19.7;22.0	18.4	17.3;19.5	2.4 (11.7)
**Perceived health status**					
Negative self-assessment of health	3.6	3.1;4.1	4.3	3.7;4.9	-0.7 (-18.6)
**≥ 55 anos years**					
Nutritional status					
Overweight	63.0	61.7;64.4	61.5	60.3;62.8	1.5 (2.4)
Obesity	21.9	20.7;23.1	22.7	21.7;23.8	-0.8 (-3.7)
**Dietary intake**					
Consumption of unprocessed or minimally processed foods^d^	36.8	35.3;38.4	33.8	32.7;35.0	3.0 (8.1)
Consumption of ultra-processed foods^e^	9.4	8.4;10.4	9.0	8.1;9.8	0.4 (4.7)
**Physical activity and sedentarism**					
Physical activity during leisure time^f^	26.9	25.5;28.3	28.2	27.1;29.3	-1.3 (-4.8)
Physical activity during transport^f^	19.6	18.3;20.8	8.4	7.6;9.1	11.2 (57.2)
High screen time^g^	85.9	84.7;87.0	49.1	47.8;50.4	36.8 (42.8)
**Smoking and harmful alcohol consumption**					
Smokers	10.8	9.8;11.7	10.9	9.9;11.9	-0.1 (-1.3)
Harmful alcohol consumption	10.4	9.5;11.3	7.9	7.2;8.6	2.5 (23.8)
**Perceived health status**					
Negative self-assessment of health	8.2	7.4;8.9	6.7	6.1;7.3	1.5 (18.1)

a) PNS: National Health Survey; b) VIGITEL: Surveillance System for Risk and Protective Factors for Chronic Diseases by Telephone Survey; c) 95%CI: 95% confidence interval; d) ≥ 5 unprocessed or minimally processed foods in the 24 hours prior to the interview; e) ≥ 5 ultra-processed foods in the 24 hours prior to the interview; f) ≥ 150 minutes per week; g) ≥ 3 hours per day; h) Difference between the PNS and VIGITEL data; i) Absolute difference (in percentage points [p.p.]) and relative difference (in percentage) using the PNS data as the baseline.

For the 35 to 54 age group, differences in prevalence estimates were observed for overweight, at 2.8 p.p. (PNS = 65.1% vs. VIGITEL = 62.3%), physical activity during transport, at 4.8 p.p. (PNS = 21.7% vs. VIGITEL = 16.9%), high screen time, at 21.6 p.p. (PNS = 79.9% vs. VIGITEL = 58.3%) and alcohol abuse, at 2.4 p.p. (PNS = 20.8% vs. VIGITEL = 18.4%) (Table 4)

In the age group of ≥ 55 years, differences were identified for: consumption of unprocessed or minimally processed foods (3.0 p.p.; PNS = 36.8% vs. VIGITEL = 33.8%), physical activity during transport (11.2 p.p.; PNS = 19.6% vs. VIGITEL = 8.4%), high screen time (36.8 p.p.; PNS = 85.9% vs. VIGITEL = 49.1%), alcohol abuse (2.5 p.p.; PNS = 10.4% vs. VIGITEL = 7.9%) and negative health self-assessment (1.5 p.p.; PNS = 8.2% vs. VIGITEL = 6.7%). With increasing age, there was an increase in the percentage of consumption of unprocessed or minimally processed foods and negative self-assessment of health status, in parallel with a decrease in physical activity during leisure time, during transport, and of abusive consumption of alcohol for both surveys (Table 4).

## Discussion

The present study presented and compared the frequencies of the main risk and protective factors for NCDs related to lifestyle in the adult population of state capitals and the Federal District, according to PNS 2019 and VIGITEL 2019. For most indicators, the results were similar, especially when the questions and response options were similar. However, the greatest differences in prevalence estimates were identified among indicators related to physical activity and sedentary behavior (high screen time), for the entire population and most of the stratifications. Among the stratifications, attention is drawn to the higher number of indicators with differences in prevalence for younger individuals (18 to 34 years old) and for those aged ≥ 55.

The results of the present study deepen and update the comparative analysis carried out based on data from the 2013 PNS and the 2013 VIGITEL.^10^ In that investigation, 11 risk and protective factors were compared for the entire population and by sex. Of those, only three were included in the present study (smokers, abusive consumption of alcohol and recommended physical activity during leisure time), given that the monitoring of most of the other factors was discontinued in the period after the replacement of indicators. It should also be noted that the previous investigation also included indicators for which the calculation methodology already indicated a difference between the surveys and, therefore, for which there was not any expectation of agreement.

The increase in agreement between the studies prevalence obtained may reflect the effort to harmonize the main health surveys investigating risk and protective factors for NCDs. It should be noted that the dissimilarities identified can be explained, resulting from methodological differences,^11^ involving the sampling, questionnaire and approach used for data collection.^11^ ^-^ ^13^ In the comparison carried out in the present study, two important characteristics should be highlighted: the study population and the data collection method. While the PNS starts from a registry of households in the country to conduct face-to-face household interviews, VIGITEL relies on samples of household landline telephone records provided by the main telephone operators in the country to carry out telephone interviews. Therefore, VIGITEL already starts with a smaller study population than that of the PNS, given that the coverage of landline service in the capitals is close to 60%. Besides, in some of these, it is not possible to carry out an interview even after several attempts (the non-response rate in household surveys ranges from 15% to 20%, while for telephone surveys it ranges from 36% to 60%, depending on the methodology used to estimate and identify the effectively eligible lines).^5^ ^,^ ^14^ ^-^ ^16^ Even though statistical adjustments are applied in the form of weighting factors, these are not always sufficient to correct such problems.

Previous studies, which compared data from household and telephone surveys, show similarities for most of the indicators analyzed, as is the case of the study conducted in Belo Horizonte/Minas Gerais, with data from VIGITEL, and the household study, Saúde em Beagá (Health in the area of Belo Horizonte),^17^ and in the study carried out in Campinas/São Paulo, with the ISACamp (household survey) and VIGITEL^18^ (telephone survey) databases, carried out in 2008. These were used to compare chronic health conditions and, in both studies, similar results were obtained for most of the self-reported conditions investigated.^17^ ^,^ ^18^ Such conditions, on the other hand, were not investigated in this study. The investigation of the quality of the surveys was also analyzed in locations with low telephone service coverage, as is the case of the capitals Rio Branco/Acre, in 2007 (40% coverage),^19^ and Aracaju/Sergipe, in 2008 (49% coverage).^20^ It was observed that the post-stratification process was able to correct most of the biases in the prevalence of the indicators studied^19^ ^,^ ^20^ but it did not reduce the sample bias for the indicator concerning physical activity during leisure time, for example.^19^ Differences in the results of the indicators of physical activity, also observed in the present study, may result from the different response options for the construction of the indicator (Box 1) - in the PNS, the respondent can openly report the number of hours they engaged in physical activity, and in VIGITEL, the response options are closed -, which may lead to an overestimation of the indicators of physical activity. It is also worth noting that for the indicator of sufficient physical activity during transport in the PNS, the addition of an option in the answer to the questions (addition of the answer option “club”), may have reflected in higher prevalence, when compared to VIGITEL (Box 1). Besides that, in spite of the fact that both surveys were based on self-reported information, it is commonly accepted that face-to-face interviews, especially those conducted in households, provide the opportunity to obtain better quality answers, since communication between the respondent and the interviewer takes place directly, with a greater volume of resources on the part of the interviewer.^21^

In any case, although none of the surveys used here constitute the gold standard for investigating risk and protective factors for NCDs, it is believed that their limitations do not discredit the results obtained. Household or telephone surveys are the main options for collecting data from large population samples in most countries, and self-reported information is recommended and constantly used in large health surveys to monitor NCDs and their factors.^4^

Household surveys tend to have very broad themes, especially in low- and middle- income countries, where their high cost and complex logistics make it impossible to carry out multiple surveys. As a result, they tend to form the baseline for monitoring a population. In Brazil, the PNS is the most complete health survey ever carried out, with several modules and themes, resulting in an application time ranging from 50 minutes to about 4 hours.^5^ Therefore, it is from the PNS data that the most complete health portrait of the Brazilian population is rendered. However, the high cost and logistics involved in carrying out a survey of this nature make it impossible to conduct it with great frequency for monitoring indicator trends. It is currently in its second edition, having been conducted with an interval of six years (2013 to 2019).

Thus, carrying out continuous monitoring along the years is only possible through the adoption of simpler and less expensive methods for obtaining information, as in VIGITEL. The low cost and the agility when compared to household surveys are the advantages of the surveillance system based on telephone interviews.^22^ For example, in 2006, each one of the approximately 54,000 interviews carried out by VIGITEL cost BRL 31.15,^22^ while the cost per interview of the household survey carried out by the Health Surveillance Department and the National Cancer Institute (with a questionnaire similar to the one used by VIGITEL) was around BRL 147.00.^22^ Combined with lower cost, the agility in disseminating the main results of VIGITEL stands out (available just over two months after the end of data collection),^8^ especially due to the immediate cleaning of the data soon after collection and its storage directly in electronic media.^8^

Among the limitations of the study, it should be pointed out that, despite the methodological differences, the development of the PNS questionnaire for the lifestyle module was based on the instrument already used by VIGITEL. However, issues inherent to the planning of surveys of this magnitude ended up inducing a series of differences in the questionnaires. Several possibilities must still be considered in order to find differences, such as, for example, the questions are not the same, different response options, or even an alteration in the order of the questions.^23^ The design of the present study is only sufficient to identify differences, but not their causes. Investigations in this sense would require studies with a specific design.

A second issue concerns the period of data collection for the surveys. PNS data collection started in the 8^th^ month of VIGITEL’s data collection (August 2019) and was concluded only in March 2020, about 100 days after the conclusion of the 2019 VIGITEL. Such mismatch may impact some of the prevalences that are sensitive to seasonality (mainly the indicators of physical activity).^24^ Additionally, behavioral changes induced by the onset of the COVID-19 pandemic (in early 2020) may have also been decisive for the discrepancies observed, especially in the indicators of physical activity and sedentary lifestyle. Finally, the small number of indicators validated in both surveys makes it impossible to certify which of the values would be closer to the real one, in the case of the observed discrepancies.

The interconnection between the surveys actually makes it possible to know the population's health status in detail and to identify the evolution of the main indicators. In general, both surveys showed prevalence with small differences, particularly in the case of the indicators of physical activity and sedentary behavior. However, estimates point to results in the same direction, especially in the stratification by sex and age. These results show the importance of different methodologies for monitoring the risk and protective factors of NCDs in the population, which contribute to improving the design of public policies for health promotion. 
